# Identification of new antiviral agents against Kaposi’s sarcoma-associated herpesvirus (KSHV) by high-throughput drug screening reveals the role of histamine-related signaling in promoting viral lytic reactivation

**DOI:** 10.1371/journal.ppat.1008156

**Published:** 2019-12-02

**Authors:** Jungang Chen, Lu Dai, Alana Goldstein, Haiwei Zhang, Wei Tang, J. Craig Forrest, Steven R. Post, Xulin Chen, Zhiqiang Qin

**Affiliations:** 1 Department of Pathology, Winthrop P. Rockefeller Cancer Institute, University of Arkansas for Medical Sciences, Little Rock, Arkansas, United States of America; 2 Departments of Diagnostic Sciences, School of Dentistry, Louisiana State University Health Sciences Center, New Orleans, Louisiana, United States of America; 3 State Key Laboratory of Virology, Wuhan Institute of Virology, Chinese Academy of Sciences, Hubei, China; 4 Department of Microbiology & Immunology, University of Arkansas for Medical Sciences, Little Rock, Arkansas, United States of America; 5 Guangdong Key Laboratory of Virology, Institute of Medical Microbiology, Jinan University, Guangzhou, China; University of Washington, UNITED STATES

## Abstract

Kaposi’s sarcoma-associated herpesvirus (KSHV) causes several human cancers, such as Kaposi’s sarcoma (KS) and primary effusion lymphoma (PEL). Current treatment options for KSHV infection and virus associated diseases are sometimes ineffective, therefore, more effectively antiviral agents are urgently needed. As a herpesvirus, lytic replication is critical for KSHV pathogenesis and oncogenesis. In this study, we have established a high-throughput screening assay by using an inducible KSHV+ cell-line, iSLK.219. After screening a compound library that consisted of 1280 Food and Drug Administration (FDA)-approved drugs, 15 hit compounds that effectively inhibited KSHV virion production were identified, most of which have never been reported with anti-KSHV activities. Interestingly, 3 of these drugs target histamine receptors or signaling. Our data further confirmed that antagonists targeting different histamine receptors (HxRs) displayed excellent inhibitory effects on KSHV lytic replication from induced iSLK.219 or BCBL-1 cells. In contrast, histamine and specific agonists of HxRs promoted viral lytic replication from induced iSLK.219 or KSHV-infected primary cells. Mechanistic studies indicated that downstream MAPK and PI3K/Akt signaling pathways were required for histamine/receptors mediated promotion of KSHV lytic replication. Direct knockdown of HxRs in iSLK.219 cells effectively blocked viral lytic gene expression during induction. Using samples from a cohort of HIV+ patients, we found that the KSHV+ group has much higher levels of histamine in their plasma and saliva than the KSHV- group. Taken together, our data have identified new anti-KSHV agents and provided novel insights into the molecular bases of host factors that contribute to lytic replication and reactivation of this oncogenic herpesvirus.

## Author summary

As a major oncogenic human herpesviruses, KSHV infection causes several cancers mostly seen in immunocompromised patients. Currently, effective antiviral treatments are still lacking. The “old drugs, new tricks” approach may serve as a feasible strategy for high-throughput screening of novel agents against lytic replication of KSHV. Here we screened an FDA-approved drug library and identified 15 new anti-KSHV agents. Interestingly, several of these candidates target histamine receptors, implying the involvement of histamine-related signaling in lytic replication and reactivation of KSHV. This involvement was directly demonstrated using antagonists and agonists specific for individual histamine receptors as well as RNAi. The downstream signaling pathways required for histamine-mediated promotion of KSHV lytic replication was also identified. Clinical data from a cohort of HIV+ patients confirm the relevance and elevation of histamine in the microenvironment of HIV+/KSHV+ patients. Thus, our findings provide new clues for developing anti-KSHV treatments, and identify novel mechanisms through which histamine and related signaling pathways function as important host factors facilitating KSHV lytic reactivation and pathogenesis.

## Introduction

Kaposi’s sarcoma-associated herpesvirus (KSHV), also named human herpesvirus 8 (HHV-8), is the etiologic agent of Kaposi’s sarcoma (KS), primary effusion lymphoma (PEL), and multicentric Castleman’s disease (MCD) [1,2,3]. KS is an endothelial-originated multicentric malignant neoplasm found in immunosuppressed patients, and most frequently in patients infected with HIV [1,4]. In contrast, PEL is a rare and aggressive B-cell non-Hodgkin's lymphoma that typically presents as a lymphomatous effusion without forming a solid mass [5]. MCD is also a B-cell lineage disorder with specific characteristics of cytokine excess and viral lytic activation [6]. Current therapeutics for KSHV-associated malignancies are not completely efficacious and have significant adverse side effects [7,8]. Therefore, the identification of more effective and safer anti-KSHV agents is urgently needed.

KSHV belongs to the human γ-herpesvirus subfamily and has a large genome of approximately 170 kb that encodes about 90 annotated open reading frames (ORFs) [9]. Similar to many other human herpesviruses, KSHV has two alternating life-cycle programs following primary infection in host cells, the latent and lytic phases, which are characterized by different patterns of viral gene expression [10,11]. During latency, viral genomes persist as circular episomes with no progeny virion production and only a limited number of latency-associated genes expressed, including LANA, vFlip and vCyclin [12]. Once entering the lytic phase, almost all viral genes are highly expressed, followed by genomic DNA replication and mature virion release [13]. A key characteristic of the KSHV lytic phase is that most viral genes are expressed in an orderly fashion based on the time of expression, defined as immediate-early (IE), early and late genes [14]. Most cells become latently infected following primary KSHV infection, with only a small population of cells undergoing spontaneous lytic replication [15]. However, recent findings reveal that KSHV lytic replication also plays a pivotal role in the initiation and progression of virus-associated cancers [13,16].

Several studies demonstrate that lytic replication contributes to viral oncogenesis through the inflammation caused by long-term persistent viral replication and cytokine production, providing a pro-tumor microenvironment for tumor initiation in a paracrine manner and the expression in cells of lytic viral gene products that manipulate cell cycle and directly regulate immune responses to avoid immunologic surveillance [10,17,18,19]. Meanwhile, case reports and retrospective studies have also pointed out the benefit of antiviral therapy, specifically targeting the KSHV lytic replication cycle in the treatment and prevention of KSHV-associated diseases [20]. For example, treatment with Ganciclovir (GCV) and Foscarnet (PFA) to block the viral DNA polymerase during lytic replication were shown to prevent KS in KSHV-seropositive transplant recipients [21]. In addition, GCV has been reported to be effective in MCD patients with active KSHV replication [22]. Knowing that KSHV lytic replication is important in the development of virus-associated diseases, makes screening and identifying new antiviral agents targeting viral lytic replication critical for developing new effective therapies. Here, we sought to screen for novel anti-KSHV agents using a high-throughput screening strategy applied to a library consisting of 1280 Food and Drug Administration (FDA)-approved drugs. Our screening finally identified 15 hit compounds with significant anti-KSHV activity. Additional mechanistic studies demonstrated the involvement of histamine, histamine receptors and related signaling pathways in KSHV lytic replication and reactivation.

Histamine receptors (H1R-H4R) are a class of G protein–coupled receptors (GPCRs) that bind histamine as their primary endogenous ligand [23]. Histamine, a biogenic amine, is mainly synthesized and released by mast cells (MCs) [24]. Interaction of histamine with its receptors plays a significant role in the development of various allergic diseases through regulating inflammation and immune responses [25]. Many studies showed that the transduction of biological activities by histamine receptors involve various downstream signaling pathways, including MAPK, PI3K/Akt, NF-κB [26,27,28]. Interestingly, histamine receptor expression is also detected in many tumor cells, making them sensitive to histamine stimulation, and histamine has been found to be a bivalent regulator for tumor development [29]. More importantly, MCs were recently reported to be one of the major pro-inflammatory cells within KS lesions, and the elevated circulating levels of MC granule contents, such as tryptase and histamine metabolites, were found in plasma samples from KS patients [30]. In the same study, the authors reported that one patient with clinical signs of extensive MC activation was treated with antagonists of MC pro-inflammatory mediators, which resulted in a rapid and durable regression of AIDS-KS lesions. These data suggest that MC secreted products are important components of KS tumor microenvironment, although the roles of histamine-related signaling in regulation of KSHV infection and replication are still unclear.

## Results

### Optimization and validation of an *in vitro* system for anti-KSHV drug screening

**Fig 1:**
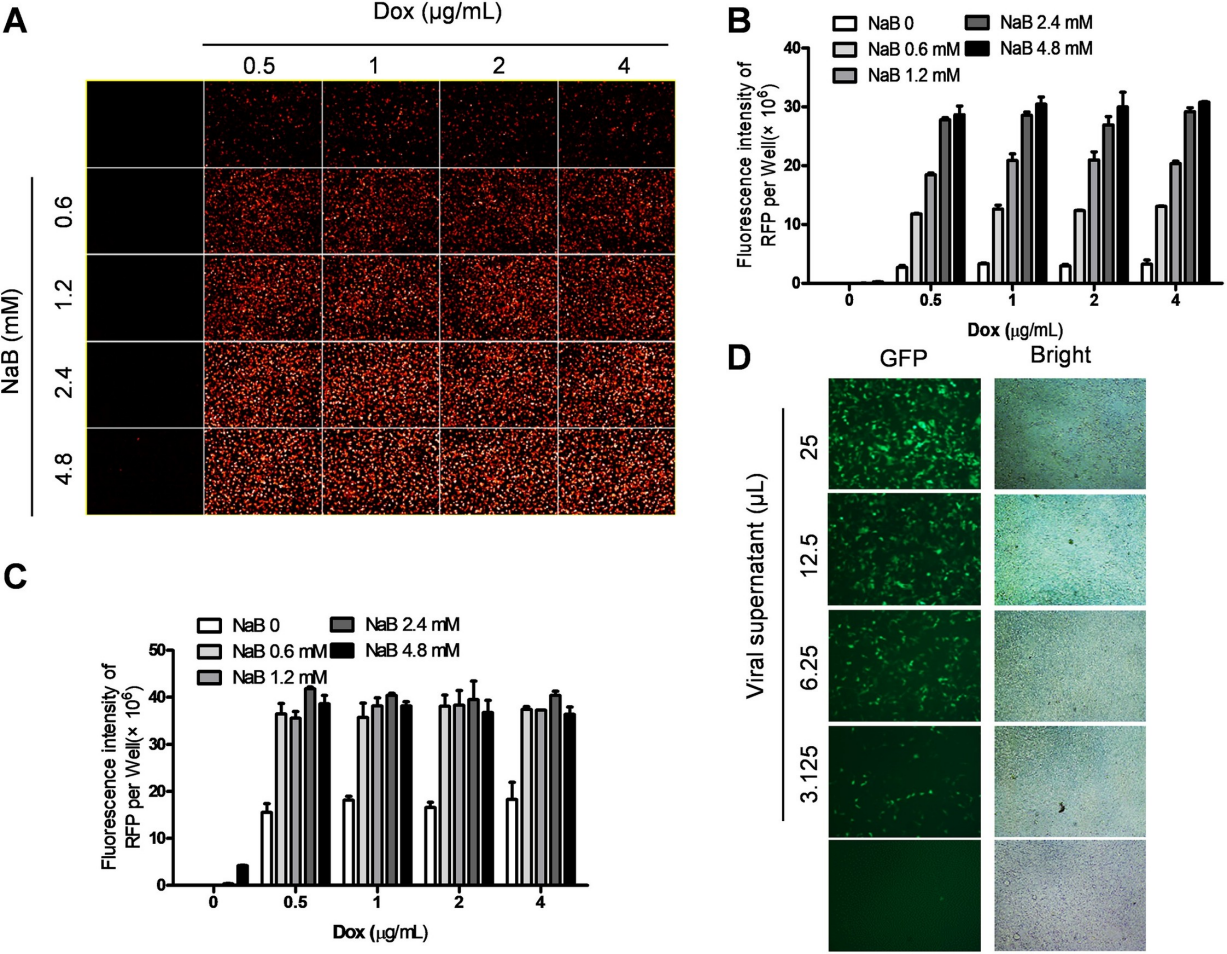
Optimization and validation of an *in vitro* system for anti-KSHV drug screening. (**A**) The iSLK.219 cells were induced by doxycycline (Dox) or sodium butyrate (NaB) alone or the combination, then RFP expression (representing viral lytic reactivation) was detected by the Operetta High-Content Screening (HCS) System at 24 h post-induction. (**B-C**) RFP expression at 24 h (B) and 48 h (C) post-induction was quantified using Harmony3.5 software. Error bars represent S.D. for 3 independent experiments. (**D**) The viral supernatants from iSLK.219 cells treated by 1 μg/mL Dox in combination with 1.2 mM NaB were collected to infected naïve HEK293T cells, then GFP expression was detected using the HCS system at 48 h post-infection.

To identify new antiviral agents against KSHV lytic replication, the human iSLK.219 cell line was used as a screening model. iSLK.219 carries a recombinant rKSHV.219 virus encoding a constitutive GFP reporter and an RTA-inducible RFP reporter in the viral genome, thereby facilitating the monitoring of viral maintenance and lytic reactivation. Previous reports demonstrated that iSLK.219 cells are suitable for anti-KSHV drug screening [31,32]. To optimize conditions for the induction of lytic replication, iSLK.219 cells were exposed to increasing concentrations of doxycycline (Dox) (up to 4 μg/mL) and/or sodium butyrate (NaB) (up to 4.8 mM), then RFP expression was detected at 24 h and 48 h post-induction using the Operetta High-Content Screening (HCS) System. We found that at 24 h post-induction, both Dox and NaB induced RFP expression, but the RFP signal induced by NaB alone was weaker than Dox alone. NaB addition synergistically promoted RFP expression by Dox in a dose-dependent manner (**Fig 1A and 1B**). At 48 h post-induction, the level of RFP expression showed minor differences among the different concentration groups except for the Dox alone group (**Fig 1C**), indicating that the effects of induction plateau at this time point. Based on these data, 1 μg/mL Dox in combination with 1.2 mM NaB was chosen as the optimal lytic-induction condition for subsequent antiviral screening. This treatment also promoted the production of mature virions as detected using an infectivity assay in which increasing doses of supernatants from induced iSLK.219 cells were used to infect HEK293T cells. As shown in **Fig 1D**, the GFP signal in HEK293T cells demonstrates successful production of infectious virions.

### High-throughput screening identifies new antiviral agents against KSHV lytic replication and virion production

**Fig 2:**
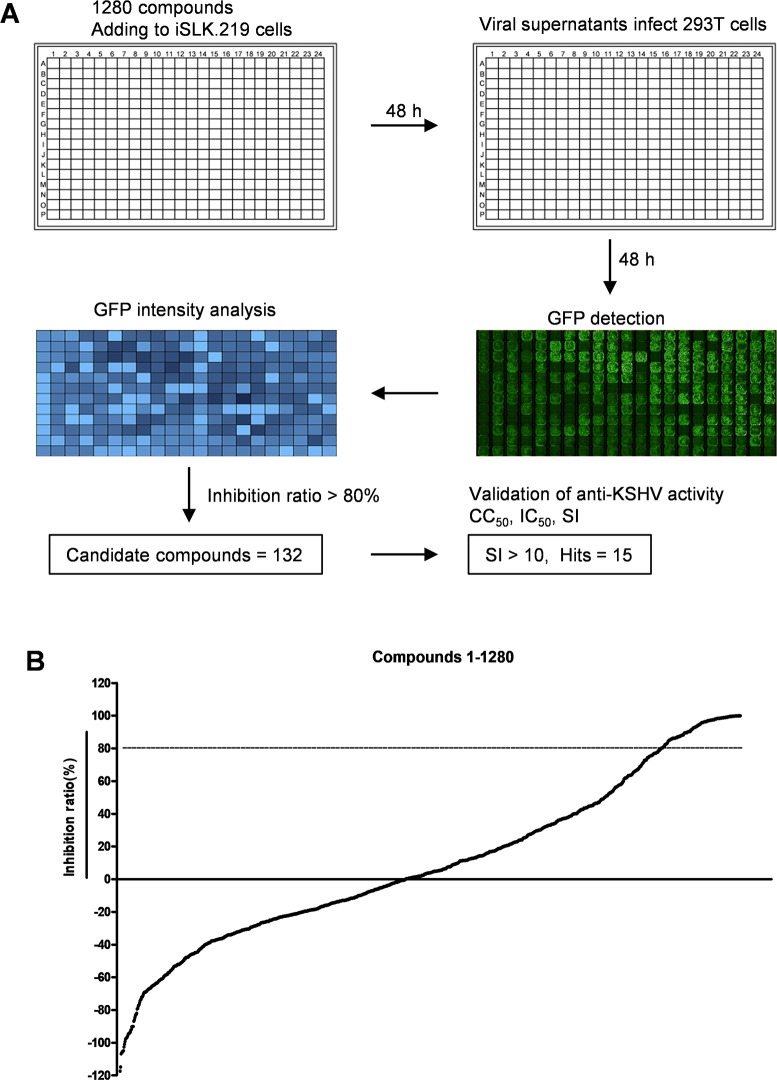
High-throughput drug screening of new agents effectively blocking infectious virion production. (**A**) Diagrams of high-throughput drug screening. The compounds in source plates were delivered at 10 μM (final concentration) to 384-well plates using Echo system, then iSLK.219 cell suspension including Dox and NaB were added. At 48 h post-induction, the supernatants were collected to infect naïve HEK293T cells seeded in new 384-well plates. The GFP expression (representing infected cells) was detected and quantified with the HCS system at 48 h post-infection. Images from nine fields per well were digitized to determine the total intensity using the Harnomy3.5 software. Data were normalized as the relative fluorescence intensity or inhibition rate compared to the DMSO control. The compounds with >80% of inhibition rate were selected to determine their IC_50_, CC_50_ and SI (CC_50_/IC_50_) as described in Methods. (**B**) Primary screening results of 1280 FDA-approved drugs against KSHV, which were arranged in order of inhibition rate.

We next sought to identify new antiviral agents using a high-throughput screening strategy from a library that consisted of 1280 FDA-approved drugs. Compounds were added to lytically-induced iSLK.219 cells in 384 well-plates, then the supernatants were collected at 48 h post-induction to infect naïve HEK293T cells. At 48 h post-infection, GFP expression was detected and fluorescence intensity was analyzed by the Operetta HCS System (**Fig 2A**). After primary screening (**Fig 2B**), 132 of these compounds were identified as potential antiviral agents with an inhibition ratio >80%. To further validate the antiviral activities of these compounds, the standard evaluation indexes, CC_50_, IC_50_ and selective index (SI, equal to CC_50_/IC_50_) were measured and calculated. Based on the dose-dependent curves of KSHV inhibition and the cytotoxicity in iSLK.219 cells, 15 compounds with SI >10 were finally selected as “hit” compounds (**Table 1**). The potential targets of these compounds were also listed in Table 1.

**Table 1:** Anti-KSHV activity of hit compounds.

Compounds	CC_50_^c^(μM)	Virion production^a^		RFP expression^b^		Targets
		IC_50_^d^(μM)	SI^e^	IC_50_ (μM)	SI	
Monobenzone	>360	0.72	>500	1.48	>243.2	Tyrosinase.
Spironolactone	300	1.07	280	7.0	42.9	Corticosteroids.
Oxibendazole	132.6	1.5	89.4	-	-	Polymerase.
Oxaliplatin	88	1.5	58.6	15.4	5.7	DNA synthesis.
Arecoline	360	6.7	53	10.6	34	Agonists of Muscarinic receptor and Nicotinic acetylcholine receptor.
Hycanthone	40	1.4	29.4	7.1	5.7	DNA topoisomerases.
Acrisorcin	23	0.8	28.7	-	-	Tyrosyl-DNA Phosphodiesterase 1.
Phenothiazine	103.8	4	26	-	-	Antagonists of Histamine receptors, Dopamine D2 receptor, Adrenergic α receptor, Serotonergic 2C receptor, and Muscarinic receptor.
Paroxetine	30	1.4	21.3	13.3	2.3	Selective serotonin reuptake inhibitor.
Protriptyline	72.5	3.7	19.6	4.4	16.5	Inhibitors of Histamine 1 receptor, Muscarinic receptor, and Adrenergic α1 receptor; Inhibiting reuptake of Serotonin and Norepinephrine.
Cyproheptadine	106.8	6.87	15.6	-	-	Antagonists of Histamine 1 receptor and serotonin receptor, 5-HT2 and 5-HT1C.
Arsenic trioxide	65.8	4.4	15	-	-	Thioredoxin reductase.
Fulvestrant	65	4.6	14.1	-	-	Steroid estrogen receptor antagonist.
Manidipine	55.2	4.2	13.1	-	-	Calcium and aldosterone antagonist.
Mefloquine	30	3	10	-	-	Cholinesterase.

a. Antiviral effects were assessed by viral infectivity assays

b. Antiviral effects were assessed by RFP expression in iSLK.219 cells

c. The CC_50_ represents the 50% cytotoxic concentration

d. The IC_50_ represents the 50% inhibitory concentration

e. SI = CC_50_/IC_50_

We further investigated whether these compounds inhibited lytic reactivation by detecting RFP expression (which represents early lytic reactivation) in iSLK.219 cells using the HCS System. Our results indicated that 7 of the 15 antiviral compounds mentioned above showed significant inhibitory effects on KSHV early lytic reactivation (**Table 1**), while the other 8 compounds may target other steps such as processes involved in assembly and egress [33].

### The anti-KSHV activity of histamine receptors antagonists

**Fig 3:**
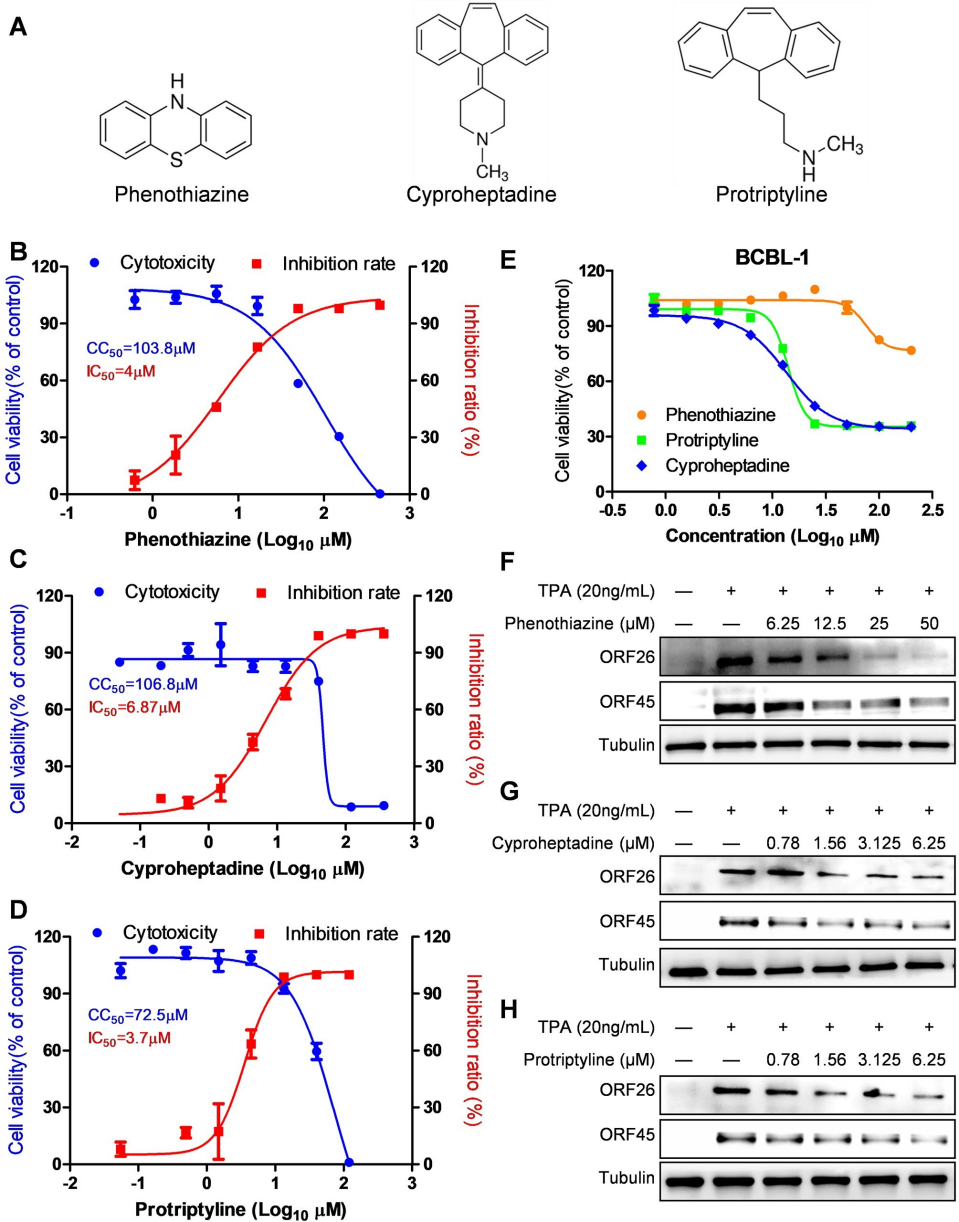
Anti-KSHV activity of 3 compounds targeting histamine receptors. (**A**) The structures of 3 compounds, Phenothiazine, Protriptyline and Cyproheptadine. (**B-D**) The lytically-induced iSLK.219 cells were exposed to these compounds at indicated concentrations, then the cytotoxicity and antiviral activities were detected as described in Methods. (**E-H**) The TPA-induced BCBL-1 cells were exposed to these compounds at indicated concentrations, then the cytotoxicity and the expression of representative lytic genes were detected as described in Methods. Error bars represent S.D. for 3 independent experiments. Representative blots from one of two independent experiments were shown.

**Fig 4:**
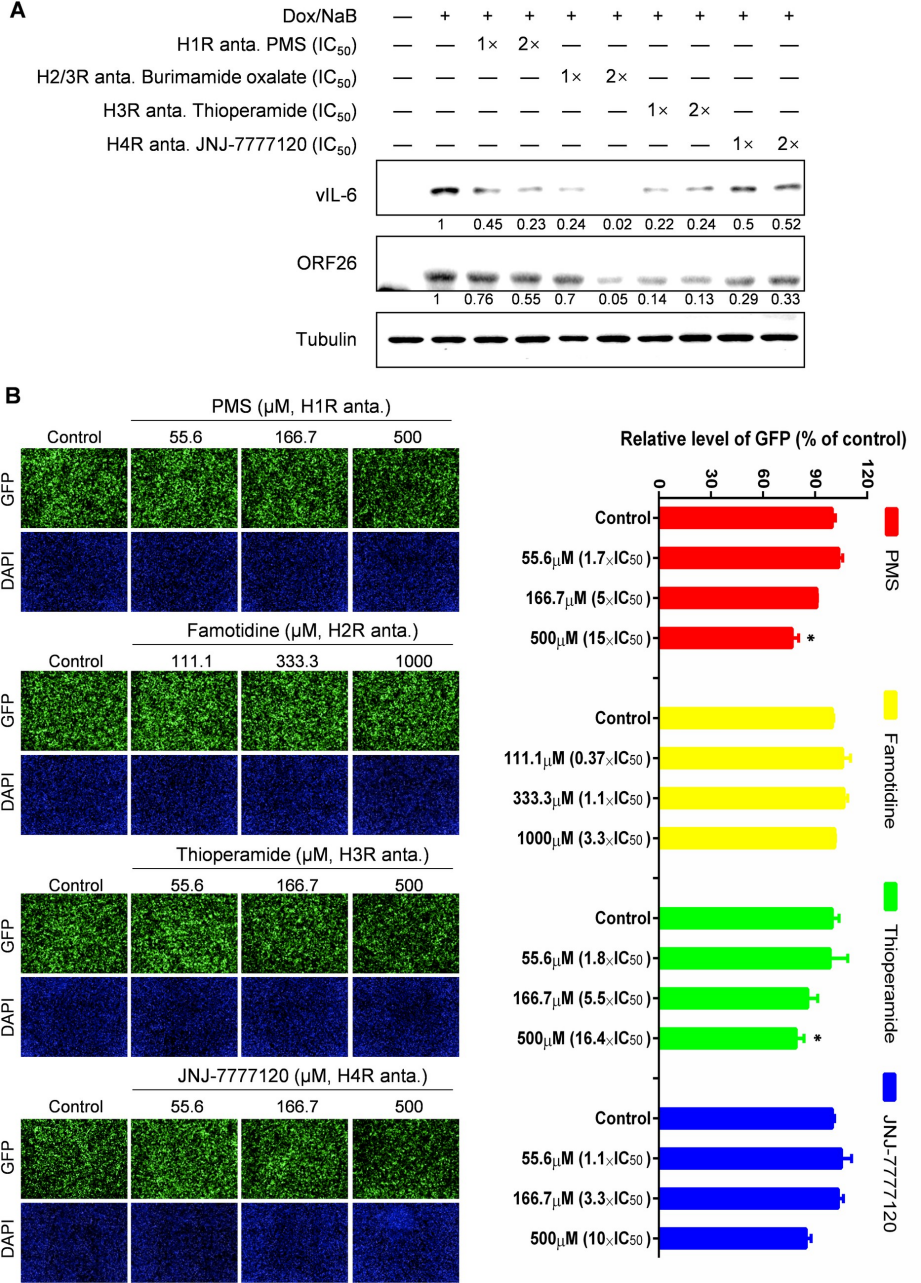
Blocking histamine receptors has minimal effects on KSHV entry. (**A**) The lytically-induced iSLK.219 cells were treated by the antagonists of different histamine receptors (H1R-H4R) at non-cytotoxic concentrations (IC_50_ and 2×IC_50_) for 48 h, then the expression of representative lytic protein, ORF26 and vIL-6, was detected by Western blot. Representative blots from one of two independent experiments were shown. Tubulin was used for loading controls. Numbers represent immunoreactivity relative to induced cells control as quantified using Image-J software. (**B**) HEK293T cells were infected by rKSHV.219 in combination with H1R-H4R antagonists using a spinoculation protocol as described in Methods, then the supernatants were removed and replaced with fresh medium. After 48 h, GFP expression was detected using the HCS system. Nine image fields per well were recorded and the associated fluorescence intensity per well was calculated using the Harmony3.5 software. Data were normalized as the fold change compared to the DMSO control. Representative images from one of two independent experiments were shown. Error bars represent S.D. for 9 image fields per well, * = p<0.05 (*vs* Control).

Interestingly, 3 of 15 hit compounds with anti-KSHV activities, Phenothiazine, Protriptyline and Cyproheptadine (**Fig 3A–3D**), all exhibit overlapping inhibitory effects on histamine receptors. Moreover, each of these 3 compounds displayed similar antiviral activities at non-cytotoxic concentrations in induced KSHV+ PEL cell line, BCBL-1 (**Fig 3E–3H**). Based on these results, we hypothesized that blocking histamine receptors inhibits KSHV lytic replication. To test this, we examined the antiviral activities of classic antagonists targeting different histamine receptors (H1R-H4R) on induced iSLK.219 cells. As shown in **Table 2** and **S1 Fig**, each of the tested antagonists of H1R, H3R and H4R effectively inhibited KSHV virion production from induced iSLK.219 cells (SI >6). It is noteworthy that all four antagonists of H3R tested (Clobenpropit, Conessine, Thioperamide, Tiprolisant) displayed potent anti-KSHV activity with SI >20. In contrast, only one of the H2R antagonists (Burimamide oxalate–which is actually a mixed H2R/H3R antagonists), displayed anti-KSHV ability with SI >6, while other H2R antagonists tested exhibited an SI <3 against KSHV, implying that blocking H2R may have less effect on KSHV lytic reactivation from iSLK.219 cells. To exclude the possibility that antiviral activities of these antagonists are due to decreasing viral entry or a virucidal effect, we evaluated viral lytic gene expression from induced iSLK.219 cells treated with representative antagonists under non-cytotoxic concentrations. Our results indicated that all representative antagonists, Pyrilamine maleate salt (PMS, H1R antagonist), Burimamide oxalate (H2R/H3R antagonist), Thioperamide (H3R antagonist) and JNJ-7777120 (H4R antagonist), significantly blocked ORF26 and vIL-6 expression under the concentrations of their IC_50_ and 2×IC_50_, respectively (**Fig 4A**). Next, we used rKSHV.219 virions to infect HEK293T cells in the presence of four representative antagonists of histamine receptors (H1R-H4R) using a spinoculation protocol. Our results showed that, regardless of histamine receptor subtype specificity, all of the antagonists at their IC_50_ concentrations displayed almost no inhibitory effects on relative GFP levels. PMS, Thioperamide, and JNJ-7777120 showed a minimal inhibitory effect (<20%) at the highest concentrations (>10×IC_50_) tested (**Fig 4B**), which may reflect the cytotoxicity. These data demonstrate that the antiviral activities of these antagonists we observed at non-cytotoxic concentrations are not the result of virucidal effects or inhibiting virus entry. Our additional data showed that selective histamine receptor antagonists, such as PMS (H1R antagonist) and Conessine (H3R antagonist), both effectively inhibited viral lytic gene expression from induced BCBL-1 cells (**S2 Fig**).

**Table 2:** Anti-KSHV activity of antagonists of histamine receptors.

Compounds	Antagonist classes	CC_50_ ^a^(μM)	IC_50_ ^b^(μM)	SI ^c^
Pyrilamine maleate salt	Histamine H1 receptor	>500	33.51	>14.9
Triprolidine hydrochloride		226.2	19.47	11.61
Diphenhydramine hydrochloride		334.2	36.33	9.20
Diphenylpyraline hydrochloride		106.8	15.97	6.69
Orphenadrine hydrochloride		8.24	1.26	6.53
Famotidine	Histamine H2 receptor	>900	300	>3
Lafutidine		>900	306.9	>2.9
Ranitidine HCL		>900	600	-
Cimetidine		>900	666	-
Burimamide oxalate	Histamine H2 and H3 receptor	>900	123.6	>7.3
Clobenpropit	Histamine H3 receptor	182	2.8	65.3
Conessine		312.7	8.31	37.6
Thioperamide		800	30.41	26.3
Tiprolisant		181.3	7.7	24
VUF6002	Histamine H4 receptor	>500	45.4	>11
JNJ-7777120		>500	50	>10

a. The CC_50_ represents the 50% cytotoxic concentration

b. The IC_50_ represents the 50% inhibitory concentration

c. SI = CC_50_/IC_50_

### Agonists of histamine receptors promote KSHV lytic reactivation and replication

**Fig 5:**
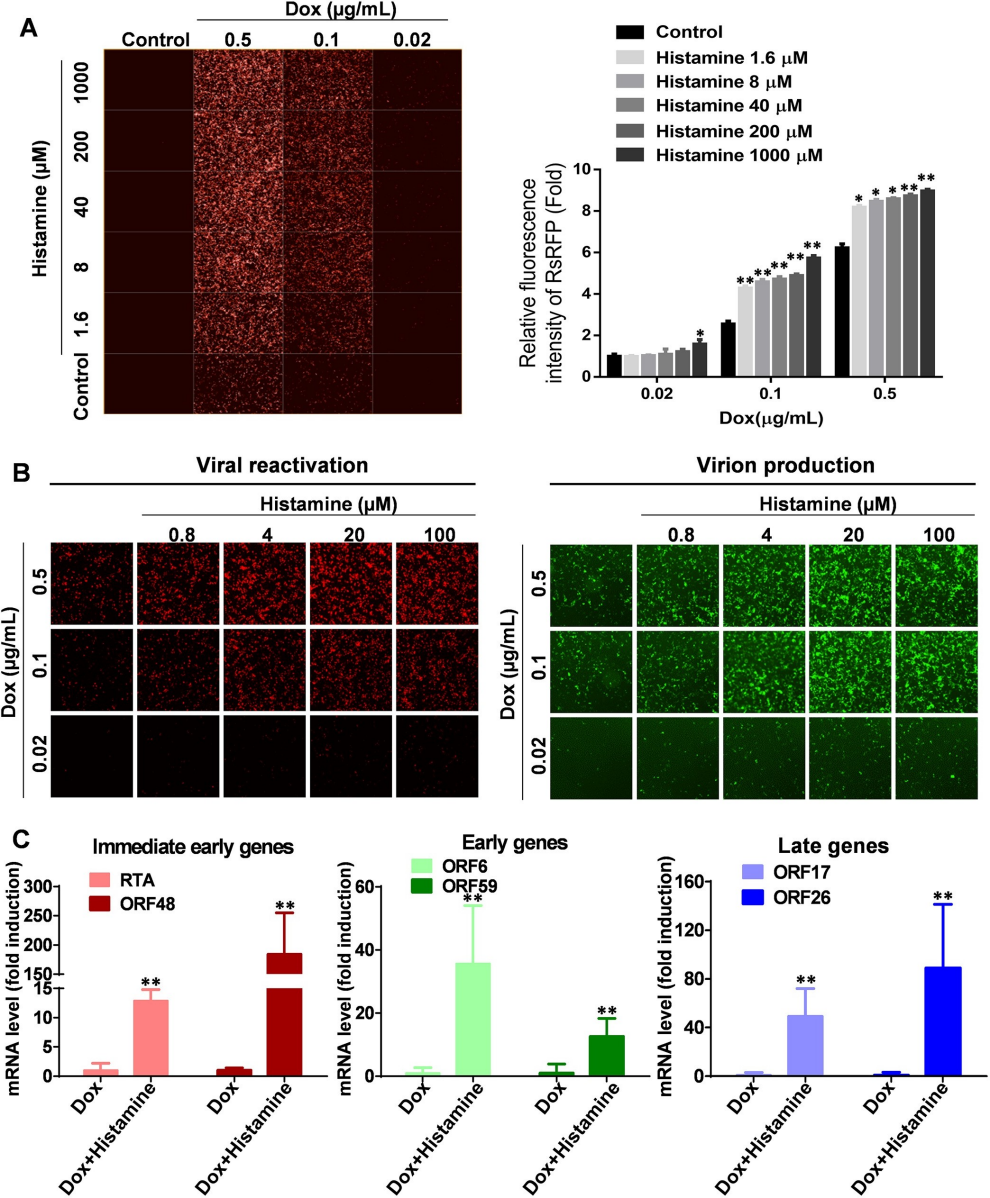
Histamine promotes KSHV lytic reactivation from iSLK.219 cells. (**A**) The iSLK.219 cells were exposed to Dox, histamine alone or the combination for 48 h, then RFP expression was detected by using the HCS system (right panel) and quantitatively analyzed using Harmony3.5 software (left panel). (**B**) The iSLK.219 cells were exposed to Dox or the combination with histamine for 48 h, then RFP expression was detected by using the HCS system (right panel). The supernatant were collected to infected naïve HEK293T cell for 48 h, then GFP expression was detected by using the HCS system (left panel). (**C**) The iSLK.219 cells were exposed to Dox or the combination with histamine for 48 h, then RNA were extracted and transcripts of viral genes were quantified by qRT-PCR. Data were normalized as the fold change compared to the DMSO control. Error bars represent S.D. for 3 independent experiments, * = p<0.05; ** = p<0.01 (*vs* Control).

To further assess the importance of histamine receptors in the lytic phase of KSHV, the ability of histamine, a universal agonist of histamine receptors, to stimulate viral lytic reactivation was tested. Both induced and non-induced iSLK.219 cells were exposed to histamine, then RFP expression was detected using the HCS System. Histidine, a precursor for histamine, was used as a negative control since it does not activate histamine receptors (**S3 Fig**). We found that histidine addition had no effect on Dox-induced RFP expression. In contrast to this, histamine synergistically enhanced Dox-induced RFP expression in a dose-dependent manner, although histamine alone treatment showed no viral induction (**Fig 5A**). We also measured lytic genes expression and virion production from iSLK.219 cells treated with both Dox and histamine. The results showed that histamine increased Dox-induced RFP expression and virion production (**Fig 5B**). Furthermore, all lytic genes that we examined, including Immediate-early (IE) genes (RTA and ORF48), Early genes (ORF6 and ORF59) and Late genes (ORF17 and ORF26), were significantly upregulated by histamine addition when compared to Dox alone groups (**Fig 5C**).

**Fig 6:**
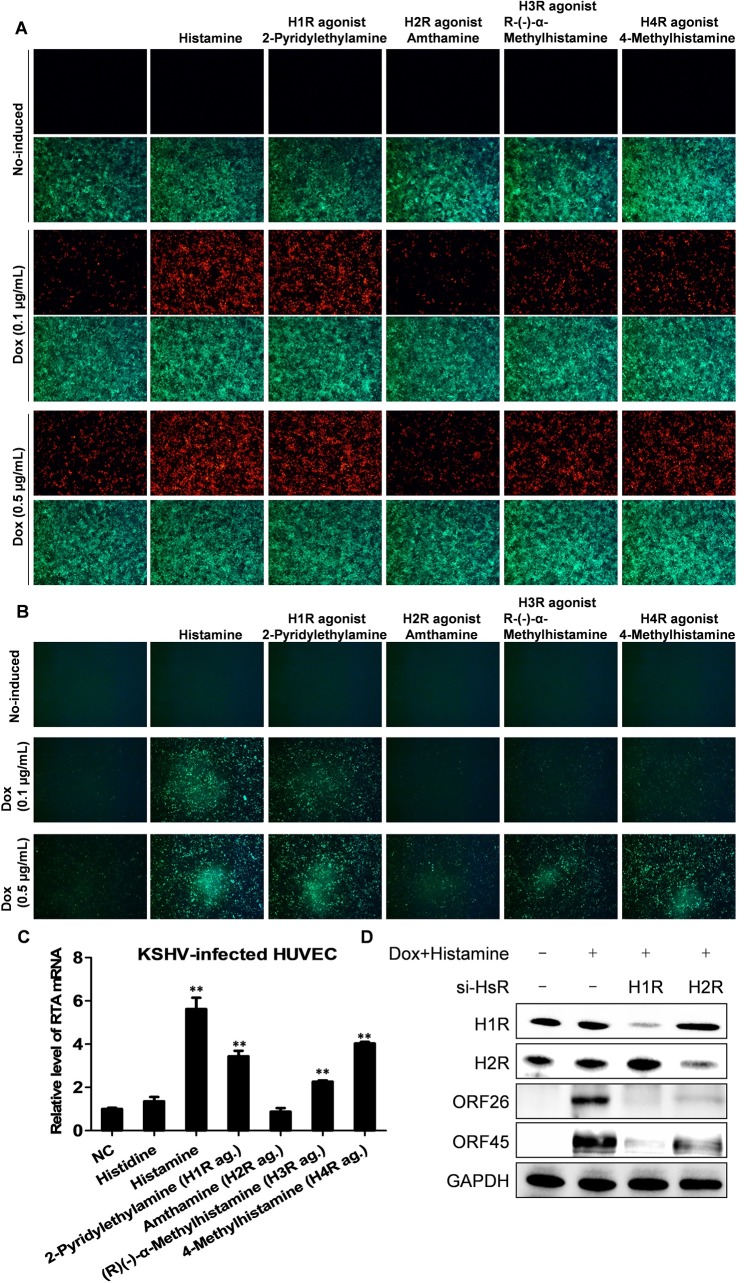
The agonists targeting selective histamine receptors promote KSHV lytic replication. (**A**) The iSLK.219 cells were exposed to Dox, the agonists of H1R-H4R alone or the combination for 48 h, then RFP expression was detected by using the HCS system. (**B)** The viral supernatants from (A) were collected to infect naïve HEK293T cells and GFP expression was detected as above at 48 h post-infection. Representative images from one of two independent experiments were shown. (**C**) The HUVEC were first infected by rKSHV.219 virions, then exposed to the desired agonists for 72 h and the transcripts of RTA were quantified by using qRT-PCR. Error bars represent S.D. for 3 independent experiments, ** = p<0.01 (*vs* NC). Data were normalized as the fold change compared to the DMSO control. (**D**) The iSLK.219 cells were first transfected with H1R-siRNA, H2R-siRNA or negative control siRNA, respectively, as described in Methods for 48 h, then induced by Dox in combination with histamine for additional 48 h. The protein expression was detected by Western blot. GAPDH was used for loading controls. Representative blots from one of two independent experiments were shown.

Since histamine is a universal agonist of histamine receptors, we further tested specific agonists of select histamine receptors, including 2-Pyridylethylamine (H1R agonist), Amthamine (H2R agonist), R-(-)-α-Methylhistamine (H3R agonist) and 4-Methylhistamine (H4R agonist). Similar to histamine, these agonists had no effects on RFP expression without Dox induction. However, the addition of 2-Pyridylethylamine, R-(-)-α-Methylhistamine or 4-Methylhistamine, significantly increased RFP expression when combined with Dox treatment, while Amthamine only showed less effect (**Fig 6A**). Furthermore, these histamine receptor agonists, especially H1R, H3R and H4R agonists, synergistically increased virion production in combination with Dox (**Fig 6B**). We also tested the effects of histamine and specific agonists on lytic reactivation from KSHV-infected HUVEC. Similar to the results in iSLK.219 cells, we observed that the addition of 2-Pyridylethylamine, R-(-)-α-Methylhistamine, 4-Methylhistamine and histamine all increased RTA expression, but not Amthamine or histidine (**Fig 6C**). To further confirm the role of histamine receptors in regulation of viral lytic reactivation, we directly targeted H1R and H2R by RNAi in iSLK.219 cells, then induced with Dox (0.5 μg/mL) and histamine (100 μM) treatment. After 48 h of induction, interestingly, we found that knockdown of either H1R or H2R blocked lytic ORF26 and ORF45 protein expression from induced iSLK.219 cells (**Fig 6D**).

### Histamine-mediated promotion of KSHV lytic replication is associated with the activation of MAPK and PI3K/Akt pathways

**Fig 7:**
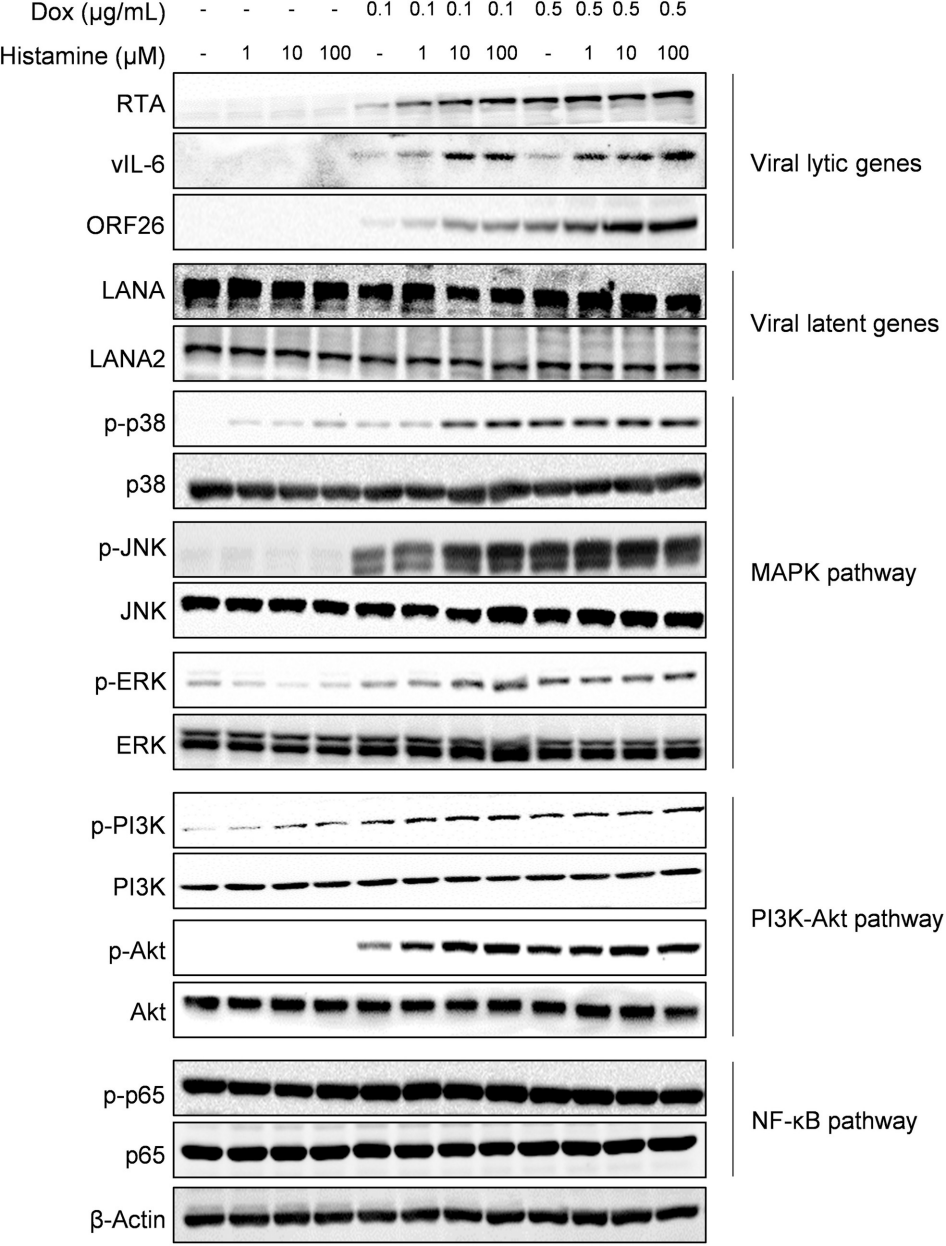
The MAPK and PI3K/Akt pathways are involved into histamine-mediated promotion of KSHV lytic replication. The iSLK.219 cells were exposed to Dox, histamine alone or the combination for 48 h. The protein expression was detected by Western blot. β-Actin was used for loading controls. Representative blots from one of two independent experiments were shown.

Histamine receptors belong to the G protein-coupled receptor family, which bind histamine to regulate intracellular signaling pathways, including PI3K/Akt, MAPK and NF-ĸB pathways [26,28]. We therefore sought to determine whether these downstream signaling pathways are involved in the histamine-mediated promotion of KSHV lytic replication. Our results confirmed that histamine treatment enhanced the expression of representative viral lytic proteins, RTA, ORF26 and vIL-6 from iSLK.219 cells in a dose-dependent manner under Dox induction, while not affecting the expression of latent proteins, such as LANA and LANA2 (**Fig 7**). Next, we found that histamine treatment led to increased activation-related phosphorylation of MAPK pathways (including p38, ERK and JNK) as well as the PI3K/Akt pathway in conjunction with Dox induction. In contrast, NF-ĸB pathway activation was not affected by histamine under the same condition (**Fig 7**). To further determine whether viral lytic replication can affect the expression of histamine receptors, iSLK.219 cells were induced by Dox in combination with NaB, then the expression of different histamine receptors was measured. Our data indicated that the expression of histamine receptors (H1R-H4R) was not affected by these induction conditions (**S4 Fig**).

**Fig 8:**
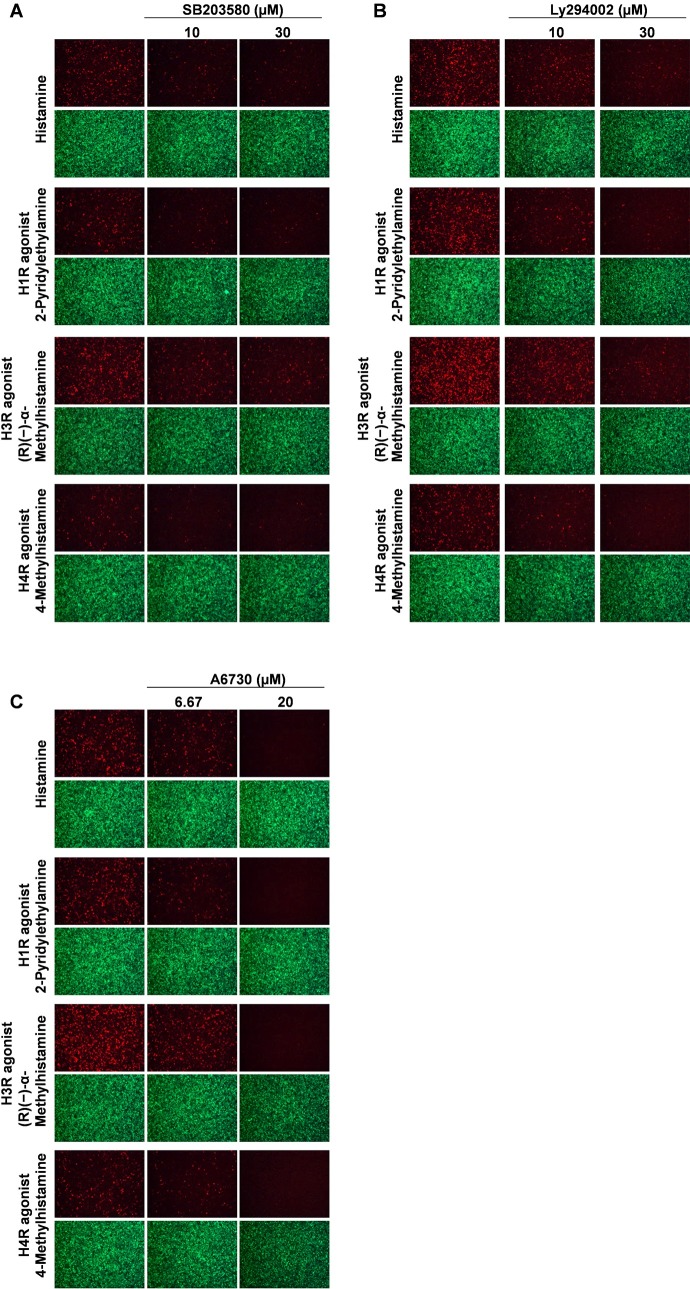
Inhibition of MAPK and PI3K/Akt pathways reduces lytic reactivation promoted by histamine and other agonists. (**A-C**) The lytically-induced iSLK.219 cells were treated with different agonists of H1R-H4R alone or in combination with pathways inhibitors, including the p38 inhibitor, SB203580 (A), the PI3K inhibitor, LY294002 (B) or the Akt inhibitor, A6730 (C). RFP expression was detected as described above. Representative images from one of two independent experiments were shown.

To examine the functional importance of MAPK and PI3K/Akt pathways in histamine and/or other agonists mediated promotion of KSHV lytic replication, we used specific pharmacological inhibitors to block these pathways. Treatment with SB203580, a MAPK-p38 inhibitor, dose-dependently reduced RFP expression from Dox-induced iSLK.219 cells exposed to histamine and/or other agonists of H1R, H3R and H4R (**Fig 8A**). Similar inhibition was observed by treating with LY294002 (a PI3K inhibitor) and/or A6730 (an Akt inhibitor) (**Fig 8B and 8C**). Taken together, these data strongly suggest that the activities of MAPK and PI3K/Akt pathways are required for augmenting KSHV lytic replication with histamine and/or histamine receptor agonists.

### Histamine levels are increased in plasma and saliva samples from HIV+/KSHV+ patients

**Fig 9:**
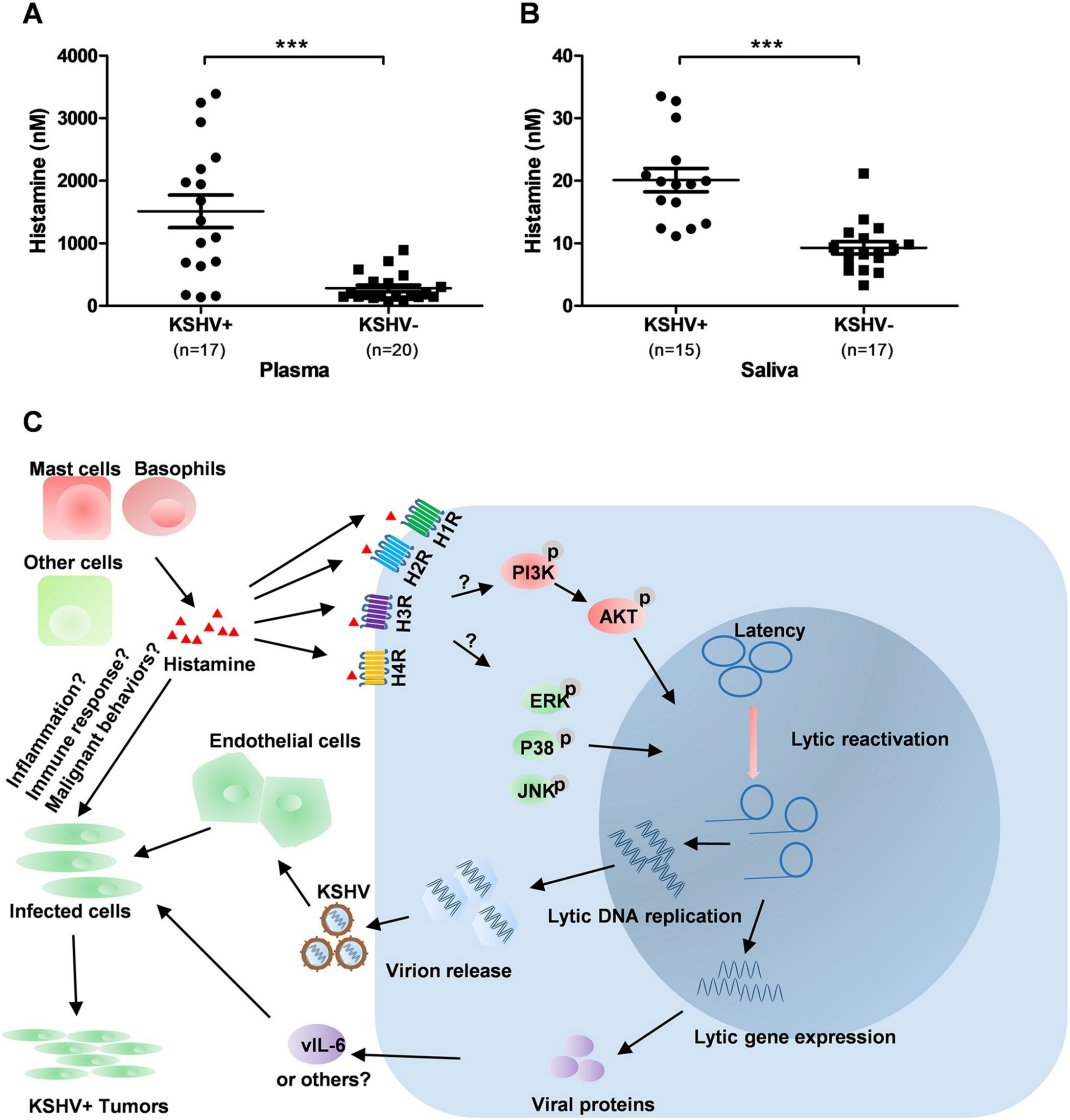
Elevation of histamine production in KSHV+ HIV-infected patients. (**A-B**) The histamine concentrations within plasma or saliva from cohort HIV-infected patients were quantified using ELISA. KSHV infection status was identified as described in Methods. *** = p<0.0001. (**C**) Proposed schematic representation of histamine related signaling promoting KSHV lytic reactivation, replication as well as viral pathogenesis.

To explore the clinical relevance of histamine production in KSHV+ HIV-infected patients, the histamine levels in plasma and saliva from a cohort of HIV-infected patients were measured using ELISA. KSHV infection status in these patients was determined as described in Materials and Methods. We found that the KSHV+ group had significantly higher histamine concentrations in their plasma and saliva (especially the former) than those from the KSHV− group of HIV-infected patients (**Fig 9A and 9B**), although saliva histamine concentrations were much lower than plasma histamine overall. There was no statistical differences in HIV viral loads or CD4 counts between the KSHV+ and KSHV- groups. These data together indicate that the upregulation of histamine is potentially related to KSHV pathogenesis in these immunocompromised patients.

## Discussion

In the current study, we employed a high-throughput screening assay to identify 15 FDA-approved drugs as new and potent anti-KSHV agents with SI>10 capable of blocking KSHV lytic replication. Moreover, we demonstrate for the first time that histamine receptors play a role in facilitating KSHV lytic reactivation. This is supported by results showing that the inhibition of most histamine receptors by antagonists reduces KSHV lytic replication, while stimulation of these receptors by agonists promotes viral lytic replication and enhances infectious virion production. Further mechanistic studies implicate the involvement of MAPK and PI3K/Akt as downstream signaling pathways triggered by histamine and/or other agonists to promote KSHV lytic replication. Taken together, our study has provided new clues for the development of therapeutic strategies against KSHV infection and related diseases.

Among the hit compounds, Monobenzone, a depigmenting agent that typically is used to eliminate melanin from skin cells for clinical treatment of people with vitiligo [34], is highly effective in inhibiting KSHV lytic reactivation and virion production with the best SI (> 500) and little cytotoxicity (CC_50_ >360 μM), suggesting its high potential to become an effective and safe drug for the treatment of KSHV infection, although to our knowledge it has never been reported to have antiviral activity. In contrast, another hit compound, Spironolactone, an aldosterone receptor antagonist used to treat heart failure, hypertension and hypokalemia [35], was previously demonstrated to have antiviral activity against several herpesviruses, including Epstein-Barr virus and Equid alpha-herpesviruses [36,37]. Not surprisingly, a cluster of compounds related to DNA synthesis or replication were also identified as hit candidates, including Hycanthone, Oxaliplatin and Oxibendazole. For example, as an anti-schistosomal drug [38], Hycanthone is an inhibitor of topoisomerase II (Top II), an enzyme that is indispensable for KSHV lytic replication [39]. Similarly, the anti-KSHV activities of Oxaliplatin, a chemotherapy drug used for treating advanced colon and rectal cancers, and Oxibendazole, a polymerase inhibitor for the treatment of intestinal helminth infections, are largely dependent on their inhibition of DNA synthesis [40,41]. Interestingly, our hit compounds also include several inhibitors targeting a series of neurotransmitter receptors, including dopamine receptors, adrenergic receptors, serotonin receptors, muscarinic receptors and histamine receptors. Although published results indicate that some neurotransmitter receptors, such as adrenoreceptors and dopamine receptors, can regulate KSHV reactivation through related signaling pathways [42,43], the functional roles of most neurotransmitters in KSHV lytic replication still remain unclear. It is conceivable that some neurotransmitters may interact with inflammatory factors to create and/or maintain a pro-lytic microenvironment for this oncogenic virus.

In the current study, we focus on the roles of histamine, its receptors and downstream signaling in KSHV lytic replication. One previous report found that histamine addition into KS-1 cells (a KSHV+ PEL cell line) induced KSHV lytic gene expression through activating PKA signaling pathway [43], but which histamine receptors were involved in this process was not explored. In the current study, we found that KSHV lytic reactivation was inhibited by targeting histamine receptors by both antagonists and RNAi, while viral replication was enhanced by histamine receptor agonists, demonstrating that histamine receptors indeed affect viral lytic replication. Interestingly, the antagonists and agonists of H2R displayed less effects on viral reactivation and replication when compared to those of other histamine receptors in iSLK.219 cells. However, we speculate that histamine receptor subtypes will exhibit differential expression on the various cells targeted by KSHV for infection, and they are probably differential among endothelial cells, epithelial cells, fibroblasts, and B cells, when compared to iSLK.219 cells. Future studies will identify which histamine receptor subtypes mediate histamine promotion of KSHV lytic reactivation in different host cells.

It is also possible downstream signaling by specific histamine receptors functions through different proteins and pathways. For example, a recent study in colorectal cancer cells demonstrated that H2R activation reduced MAPK phosphorylation to suppress their activities [44], which differs from the activating effects of other histamine receptors on MAPK pathways [44,45]. Our findings here demonstrate that the MAPK and PI3K/Akt signaling pathways are involved in the histamine-mediated promotion of KSHV lytic reactivation. Other studies report that the MAPK pathways, including p38, JNK and/or ERK, are essential for KSHV reactivation [46], and the PI3K/Akt pathway is closely related to KSHV lytic reactivation [47]. However, the underlying mechanisms for histamine receptors in regulating these signaling pathway activities in KSHV-infected cells still require further investigation.

We demonstrate here histamine levels are elevated in plasma and saliva samples from a cohort of HIV+/KSHV+ patients when compared to HIV+/KSHV- ones, which implicates histamine in the clinical progression of KSHV pathogenesis and virus-associated diseases. However, we note that plasma and saliva histamine levels (especially the latter) were lower than those we used in cell culture assays. In fact, we think the patients’ histamine levels are greatly underestimated in our test, because 1) although histamine rises much more rapidly than does serum tryptase, the short half-life of histamine, on the order of minutes, and the difficulties in handling patient samples usually preclude accurate measurements [48,49]; 2) some chemical components of mouthwash we used to collect saliva may accelerate histamine degradation. Therefore, future work will use fresh samples, including whole saliva directly collected without mouthwash or test stable metabolites of histamine such as N-methylhistamine [30].

Although our current study focuses on the role of histamine, receptors and signaling in KSHV lytic reactivation and replication, the histaminergic system may also directly contribute to viral oncogenesis. There is now overwhelming evidence supporting the significance of histamine/receptors in cancer formation and spread, producing protumor or, conversely, antitumor effects, which depend on different tumor types [50]. Interestingly, a recent study reported that the use of antagonists of MC pro-inflammatory mediators (including histamine receptors antagonists) successfully caused a rapid and durable regression of AIDS-KS lesions in one patient [30]. They also found increased plasma N-methylhistamine levels in AIDS-KS and classic KS patients when compared to healthy comparators.

In conclusion, the lacking of effective treatment for KSHV-associated malignancies, especially in immunocompromised patients, requires the discovery and development of novel and safe therapeutic strategies. In this study, we have identified 15 FDA-approved drugs with excellent anti-KSHV activities, which may offer clues that foster the development of new antiviral treatments. Moreover, we have for the first time determined the roles of histamine, histamine receptors and downstream signaling pathways in the regulation of KSHV lytic reactivation and replication (summarized in **Fig 9C**), which highlights a previously unknown virus-host interaction and potential mechanism driving the pathogenesis of this oncogenic virus.

## Materials and methods

### Cell culture and reagents

Human iSLK.219 cells (Dox-inducible SLK carrying rKSHV.219), derived from SLK cells (although a recent study reported SLK is not of endothelial origin, but is a contaminant from a known renal carcinoma cell line [51]), are latently infected with a recombinant rKSHV.219 virus and contain a doxycycline (Dox)-inducible RTA, constructed and named by Dr. Don Ganem’s lab [32]. The rKSHV.219 virus expresses both the red fluorescent protein (RFP) under the control of KSHV lytic PAN promoter and the green fluorescent protein (GFP) under the control of the elongation factor 1 promoter (EF-1α) [52]. The iSLK.219 cells can be reactivated and then to release infectious virion progeny under the condition of Dox (Sigma-Aldrich, St. Louis, MO, USA) in combination with sodium butyrate (NaB) (Sigma-Aldrich, St. Louis, MO, USA) as described previously [31]. HEK293T (Human embryonic kidney 293T) cells were purchased from American Type Culture Collection (ATCC) and cultured as recommended by the manufacturer. Body cavity-based lymphoma cells (BCBL-1, KSHV^+^/EBV^neg^) cells were kindly provided by Dr. Dean Kedes (University of Virginia) and maintained in RPMI 1640 medium with supplements (10% fetal bovine serum, 10 mM HEPES, 100 U/mL penicillin, 100 μg/mL streptomycin, 2 mM L-glutamine, 0.05 mM β-mercaptoethanol, and 0.02% sodium bicarbonate). The lytic replication in BCBL-1 was induced by 20 ng/mL 12-O-tetradecanoyl-phorbol-13-acetate (PMA/TPA) (Sigma-Aldrich, St. Louis, MO, USA) as described previously [31]. The antagonists and agonists of histamine receptors were all purchased from Sigma-Aldrich. SB203580, Ly294002 and A6730 were obtained from Selleck Chemicals (Houston, TX, USA).

### High-throughput antiviral drug screening

A compound library consisted of 1280 FDA-approved drugs were purchased from MicroSource Discovery Systems, Inc. (Gaylordsville, CT, USA) [53]. The source plates containing compounds were used to deliver 10 μM (final concentration) of each drug to 384-well screening plates using an acoustic dispensing Echo 550 instrument (Labcyte, Sunnyvale, CA, USA) and at once the iSLK.219 cells treated by inducers or not were added to these screening plates. The viral supernatants were collected at 48 h post-induction to infect naïve HEK293T cells seeded in 384-well plates by spinoculation as reported previously [31]. The supernatants were then removed and replaced with fresh medium. At 48 h, fluorescence expression per well was detected using the Operetta High-Content Screening (HCS) System (PerkinElmer, Waltham, MA). Nine image fields per well were recorded by the automated microscope-based HCS and the associated fluorescence intensity per well was calculated using the Harmony 3.5 software (PerkinElmer, Waltham, MA) [31]. Data were normalized as the fold change compared to the DMSO control. The 50% inhibitory concentration (IC_50_) for each compound was calculated from these dose-response curves using Graphpad5.0 Prism.

### Cytotoxicity assay

The cell viability under tested compound addition was assessed by CellTiter-Glo Luminescent Cell Viability Assay (Promega, San Luis Obispo, CA, USA) according to the manufacturer’s protocol as described previously [31]. The luminescent signal was measured using the Envison 2102 Multilabel Reader (Perkin Elmer). The 50% cytotoxic concentration (CC_50_) for each compound was calculated from these dose-response curves using Graphpad5.0 Prism.

### Western blot

The expression of the proteins of interest was detected by Western Blot using protein-specific antibody as described previously [54]. Antibodies for phosphor- or total-p38, ERK, JNK, p65, PI3K, and Akt were obtained from Cell Signaling Technology (Danvers, MA, USA). Antibodies for H1R-H4R were purchased from Abcam (Cambridge, MA, USA). KSHV-LANA antibody was purchased from Advanced Biotechnologies Inc (Eldersburg, MD, USA). Antibodies for KSHV-ORF26, LANA2 and ORF45 were purchased from Novus Biologicals (Centennial, CO, USA). Antibodies for vIL-6 and RTA were obtained from ABBIOTEC (San Diego, CA, USA).

### RNA interference (RNAi) assays

For RNAi assays, *H1R* or *H2R* On-Target plus Smart pool small interfering RNA (siRNA; Dharmacon) or negative control siRNA (50 nM) were delivered using the DharmaFECT transfection reagent as recommended by the manufacturer. At 48 h post-transfection, the cells were induced by Dox (0.5 μg/mL) in combination with histamine (100 μM) for additional 48 h, then protein expression was measured by Western blot.

### qRT-PCR

Transcripts of genes of interest were also measured by qRT-PCR as previously described [54]. All qRT-PCR assays were performed using a Bio-Rad CFX384 Touch Real-Time PCR detection system using the iTaq Universal SYBR Green Supermix (Bio-Rad) with viral specific primers as follows: ORF50 (Forward, 5'-CACAAAAATGGCGCAAGATGA-3' and reverse, 5'- TGGTAGAGTTGGGCCTTCAGTT-3'); ORF48 (Forward, 5'-CGGGCAAGCAAGCTGGT-3' and reverse, 5'-CCCTGGCGATTTTGGGTAC-3'); ORF6 (Forward, 5'-CTGCCATAGGAGGGATGTTTG-3' and reverse, 5'-CCATGAGCATTGCTCTGGCT-3'); ORF59 (Forward, 5'-CGAGTCTTCGCAAAAGGTTC-3' and reverse, 5'-AAGGGACCAACTGGTGTGAG-3'); ORF17 (Forward, 5'-AGATTTTTCACGGGGGCTCTGG-3' and reverse, 5'- TGGGCTGGACACTGGGTCTATTTC-3') and ORF26 (Forward, 5'-GCTCGAATCCAACGGATTTG -3' and reverse, 5'- AATAGCGTGCCCCAGTTGC-3'). The data were normalized to the β-actin housekeeping gene expression (forward, 5'-ATCGTGCGTGACATTAAGGAG-3' and reverse, 5'-GGAAGGAAGGCTGGAAGAGT-3'). “No template” (water) controls were used to ensure minimal background contamination. Using mean Ct values tabulated for each gene, and paired Ct values for *β-actin* as a loading control, fold changes for experimental groups relative to assigned controls were calculated using automated iQ5 2.0 software (Bio-Rad).

### Patients and ethics statement

The study was approved by the Institutional Review Boards for Human Research (IRB, No. 8079) at Louisiana State University Health Science Center (LSUHSC). All participated patients (adult > 18 years old) in LSUHSC HIV Outpatient (HOP) Clinic were provided written informed consent. In the current study, a total of 37 HIV+ patients with antiretroviral treatment (ART) in our HIV Outpatient (HOP) Clinic are involved. There are 16 females and 21 males, the average age is 49.4 y (range 21–67 y). The average CD4 T cell counts is 558/mL (range 35–1,773/mL), and the average HIV viral loads is 5,734 copies/mL (range 25–66,681 copies/mL).

### Plasma and saliva preparation

Whole blood was collected in heparin-coated tubes, and plasma was isolated by centrifugation. The KSHV infection status was determined by using quantitative ELISA for identifying circulating IgG antibodies to KSHV proteins (LANA and K8.1) [55,56]. To collect the whole saliva, patients rinse with mouthwash, and saliva was collected in a wide-mouth 50 mL Nalgene tube. Typical volumes range between 3 to 5 mL of mouthwash. The patients were requested to not eat or smoke prior to providing the samples.

### ELISA

The histamine concentrations within plasma or saliva from the cohort of HIV+ patients were measured by using the Histamine ELISA kit, following the manufacturer’s instructions (Abcam, Cambridge, MA, USA).

### Statistical analysis

Significance for differences between experimental and control groups was determined using the two-tailed Student's t-test (Excel 8.0).

## Supporting information

S1 Fig Anti-KSHV activity and cytotoxicity of histamine receptors antagonists. The lytically-induced iSLK.219 cells were exposed to different histamine receptors (H1R-H4R) antagonists, then their antiviral activity and cytotoxicity were measured as described in Methods. Error bars represent S.D. for 3 independent experiments.
(TIF)

S2 Fig The anti-KSHV activity of selective histamine receptors antagonists in KSHV+ BCBL-1. (**A**) BCBL-1 cells under TPA induction were exposed to antagonists of histamine receptors, PMS or Conessine for 48 h, then the cell viability was assessed by CellTiter-Glo Luminescent Cell Viability Assay according to the manufacturer’s protocol. Error bars represent S.D. for 3 independent experiments. (**B**) BCBL-1 cells under TPA induction were exposed to PMS or Conessine at the non-cytotoxic concentrations, then the protein expression was determined by using Western blot at 48 h post-induction. Representative blots from one of two independent experiments were shown.
(TIF)

S3 Fig Histidine doesn’t promote KSHV lytic reactivation from iSLK.219 cells. The iSLK.219 cells were exposed to Dox in combination with histidine at indicated concentrations for 48 h, then RFP expression (left panel) was detected and quantitatively analyzed (right panel) as described in Methods. Data were normalized as the fold change compared to the DMSO control.
(TIF)

S4 Fig Expression of histamine receptors during KSHV lytic replication. The iSLK.219 cells were exposed to Dox alone or in combination with NaB for 48 h, then the protein expression was detected by using Western blot. Tubulin was used for loading controls. Representative blots from one of two independent experiments were shown.
(TIF)
