# Supplementary figures and images for: Identification of new antiviral agents against Kaposi’s sarcoma-associated herpesvirus (KSHV) by high-throughput drug screening reveals the role of histamine-related signaling in promoting viral lytic reactivation
Source: PLoS Pathog. 2019 Dec 2;15(12):e1008156. doi: 10.1371/journal.ppat.1008156 (PMC6907871; doi:10.1371/journal.ppat.1008156)

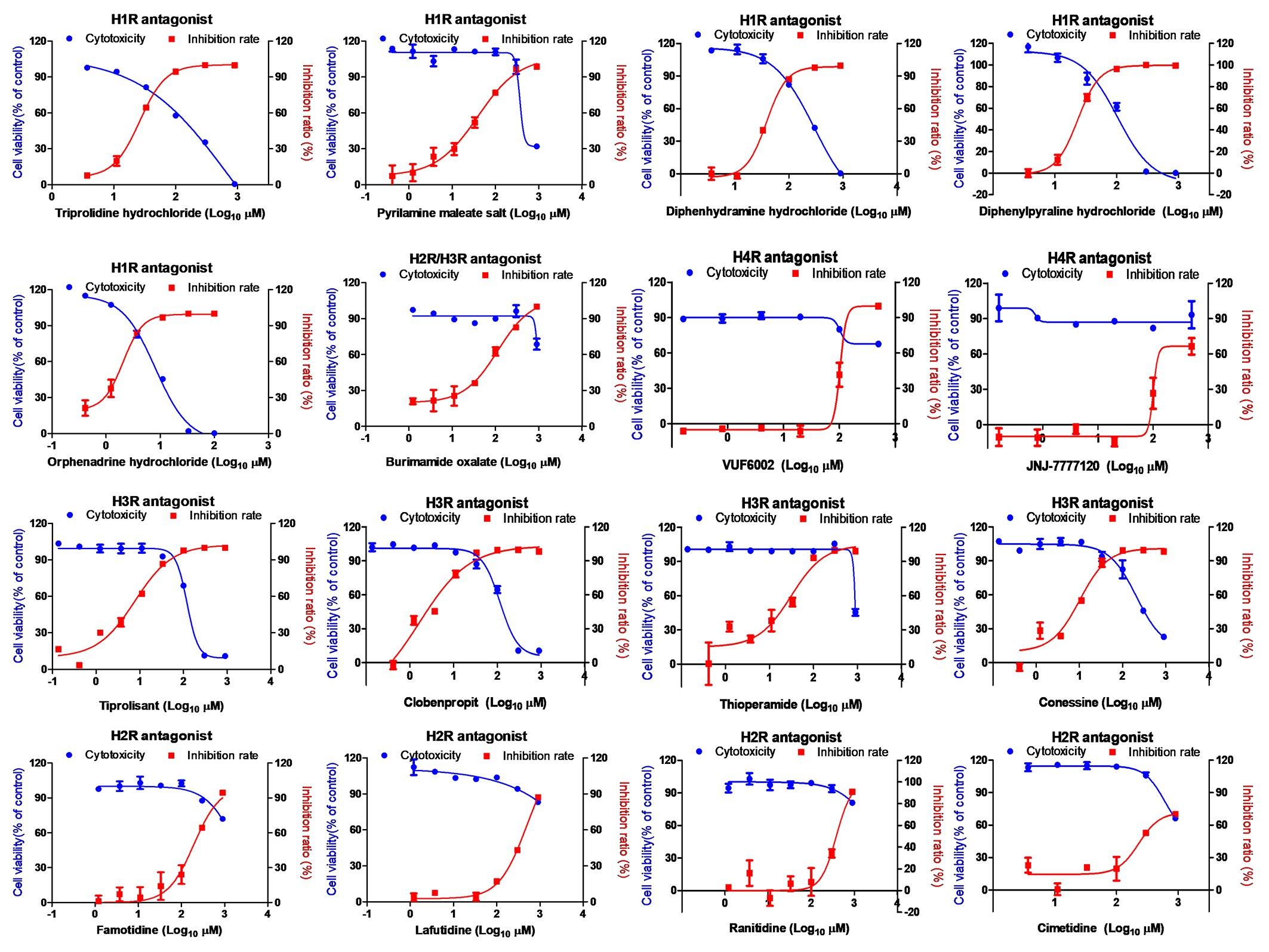

Supplement: S1 Fig — The lytically-induced iSLK.219 cells were exposed to different histamine receptors (H1R-H4R) antagonists, then their antiviral activity and cytotoxicity were measured as described in Methods. Error bars represent S.D. for 3 independent experiments. (TIF) [file ppat.1008156.s001.tif]

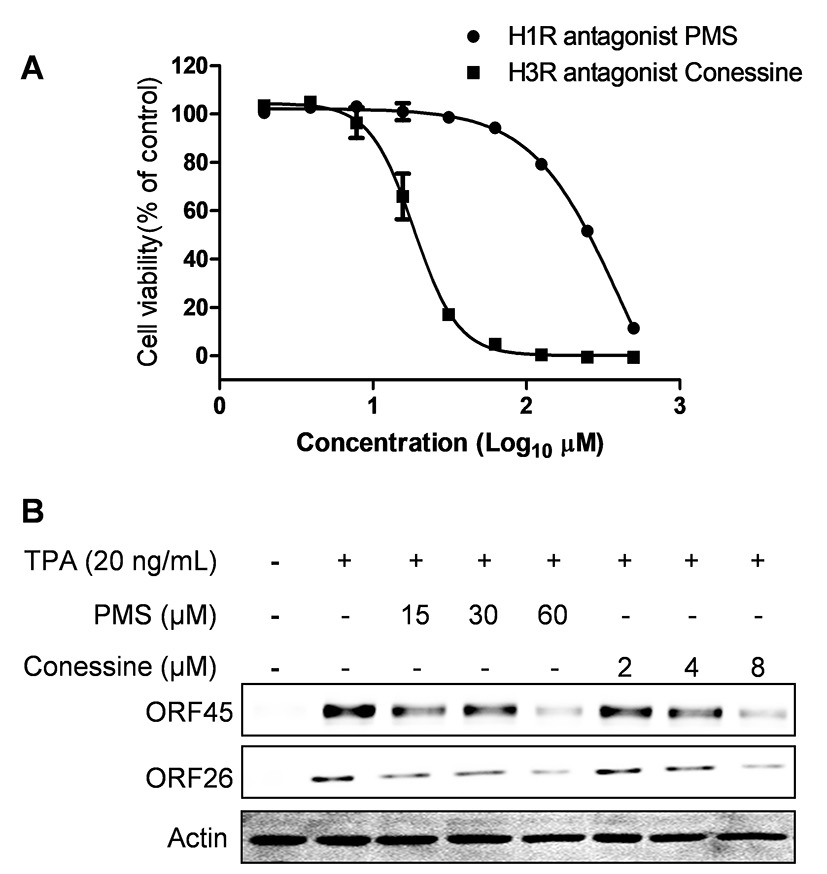

Supplement: S2 Fig — (A) BCBL-1 cells under TPA induction were exposed to antagonists of histamine receptors, PMS or Conessine for 48 h, then the cell viability was assessed by CellTiter-Glo Luminescent Cell Viability Assay according to the manufacturer’s protocol. Error bars represent S.D. for 3 independent experiments. (B) BCBL-1 cells under TPA induction were exposed to PMS or Conessine at the non-cytotoxic concentrations, then the protein expression was determined by using Western blot at 48 h post-induction. Representative blots from one of two independent experiments were shown. (TIF) [file ppat.1008156.s002.tif]

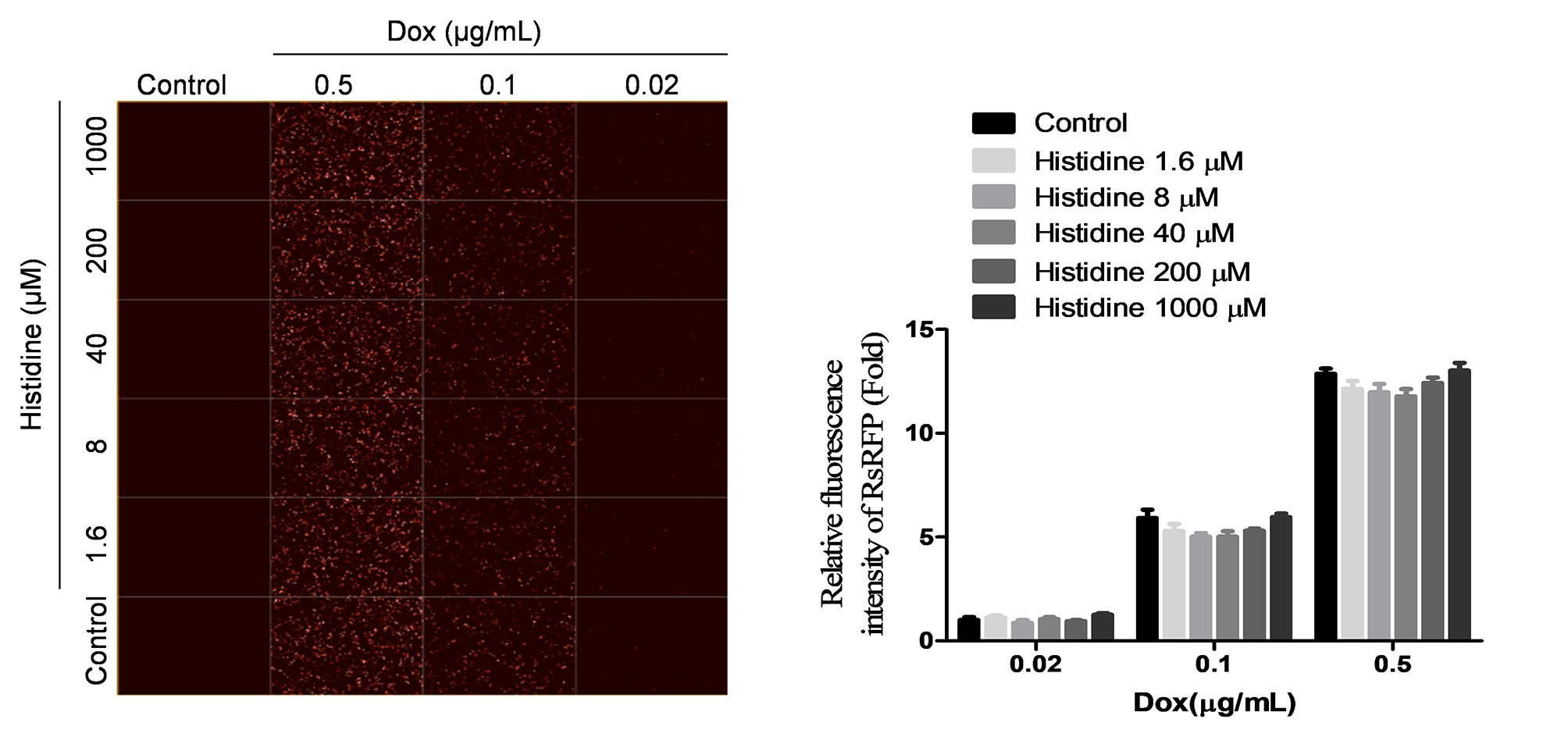

Supplement: S3 Fig — The iSLK.219 cells were exposed to Dox in combination with histidine at indicated concentrations for 48 h, then RFP expression (left panel) was detected and quantitatively analyzed (right panel) as described in Methods. Data were normalized as the fold change compared to the DMSO control. (TIF) [file ppat.1008156.s003.tif]

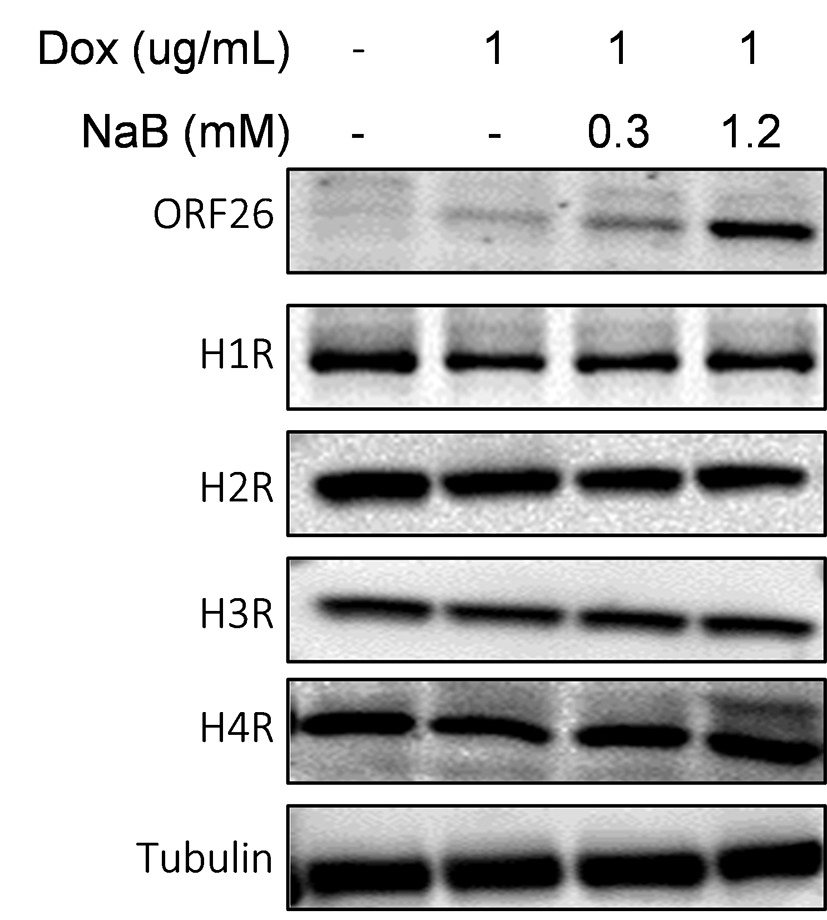

Supplement: S4 Fig — The iSLK.219 cells were exposed to Dox alone or in combination with NaB for 48 h, then the protein expression was detected by using Western blot. Tubulin was used for loading controls. Representative blots from one of two independent experiments were shown. (TIF) [file ppat.1008156.s004.tif]
